# Management of off-track Hill-Sachs lesions in anterior glenohumeral instability

**DOI:** 10.1186/s40634-023-00588-x

**Published:** 2023-03-21

**Authors:** Emilio Calvo, Cristina Delgado

**Affiliations:** Shoulder and Elbow Reconstructive Surgery Unit, Department of Orthopaedic Surgery and Traumatology, IIS-Fundación Jiménez Díaz, Hospital Universitario Fundacion Jimenez Diaz, Universidad Autonoma, Avda Reyes Católicos, 2, Madrid, 28040 Spain

**Keywords:** Shoulder, Instability, Hill-Sachs, Off-track, *Remplissage*, Latarjet, Iliac crest bone graft, Eden-Hybinette

## Abstract

Bone loss has been identified as a risk factor for recurrent shoulder dislocations or failure after soft tissue repair. Although the range for “critical” bone loss is yet to be determined, glenoid and humeral bone defects should not be regarded as independent problems, but the interaction between them during shoulder motion should be evaluated as suggested by the glenoid track concept. The glenoid track concept is now widely accepted and considered essential for making decisions about surgery. Soft-tissue procedures usually work well in patients with on-track Hill-Sachs lesions but in off-track lesions do not. In this situation additional procedures should be performed.

Different surgical options have been described to address off-track Hill-Sachs lesions, most commonly *remplissage*, Latarjet or free bone block procedures. Coracoid graft and free bone grafts convert the off-track Hill-Sachs lesion into on-track by lengthening the glenoid-track, whereas *remplissage* fill-in the humeral lesion so that it does not engage. In the setting of a Hill-Sachs lesion with little or no glenoid bone loss, *remplissage* has demonstrated satisfactory outcomes with a low complications and recurrence rate. Favorable results have been reported with glenoid bone grafting when managing isolated Hill-Sachs or bipolar lesions. Studies analyzing Latarjet and Eden-Hybinette procedures show that both procedures are safe and effective in the management of anterior glenohumeral instability. Attention should be paid to those patients with large bone defects not amenable to be restored with an isolated Latarjet that may be better addressed with an Eden-Hybinnete or adding a *remplissage* to the Latarjet procedure.

## Introduction

Glenohumeral instability is a common condition frequently affecting young and active population. Recurrent instability after a first-time anterior shoulder dislocation ranges between 7 to 80% [55]. Age below 20 years, practice of overhead or contact sports and hyperlaxity have been found to be risk factors for recurrence [10, 44]. Bone defects have also been identified as the most important predictors of recurrent instability [6].

Many techniques have been developed to treat the engaging Hill-Sachs lesion [11]. Some techniques focus in addressing the humeral defect itself including a humeral head bone grafting, humeral rotation osteotomy or filling the defect with infraspinatus tendon (*remplissage* procedure) [60]. Since the introduction of glenoid track concept, bone-grafting procedures such as the Latarjet or Eden-Hybinette procedures have been suggested as an alternative for managing engaging Hill-Sachs lesions. By bone grafting the glenoid track is restored keeping the Hill-Sachs lesion from engaging. In this manuscript the relevance of bone loss, the glenoid track concept and the different alternatives to address off-track Hill-Sachs lesions are reviewed.

## Bone loss and glenoid track

Glenoid bone loss has been reported in up to 22% after the initial dislocation and 86% of cases in recurrent instability whereas humeral bone defects (Hill-Sachs lesion) have been identified in up to 32% of patients following the first episode of dislocation and in up to 100% of patients in recurrent instability [32]. Combined glenoid and humeral bone defects (i.e. bipolar lesion) have been observed in 81% of patients with anterior glenohumeral instability [32].

In 2000, Burkhart and DeBeer [6] first recognized significant glenoid bone defects as risk factors for redislocation after a Bankart repair. They defined significant bone loss as one in which the normal morphology of the glenoid turns into an inverted pear-shaped glenoid, wider in its upper region than in the inferior one. This was observed when glenoid bone loss was superior to 25% of the articular surface. Yamamoto et al. [62] in a biomechanical cadaveric study also noted 25% as the “critical” glenoid bone loss. However, clinical studies challenge this threshold and suggest it may be lower. Calvo et al. [10] found that bone defects involving more than 15% of the glenoid diameter considerably increase the risk of recurrence after Bankart repair. This lower critical size was later supported by Shaha et al. [54], who introduced the concept of “subcritical bone loss”. They observed that, bone loss of greater than 13.5% does not result in a recurrence of dislocation, but results in poorer score at WOSI index. Regarding the humeral side, there is no consensus on the critical value of the Hill-Sachs lesion.

However, over the last 10 years it has been stated that isolated assessment of the size of the humeral and glenoid bone defects is inaccurate, and that the key is defining the interaction of these defects, specifically whether the Hill-Sachs lesion engages the anterior glenoid rim or not. This “engagement” concept was introduced by Burkhart and DeBeer [6] and divide Hill-Sachs lesions into engaging, i.e. those that when the arm is brought up into 90° of abduction and external rotation engages with the anterior glenoid rim, and non-engaging Hill-Sachs lesions. They observed that patients with engaging Hill-Sachs lesions had higher risk of recurrence and failure of soft-tissue repairs. On the latter, Yamamoto et al. [62] analyzed the dynamic interaction between the Hill-Sachs lesion and the glenoid bone defect during external rotation and abduction of the shoulder. The authors defined the contact area between the glenoid and the humerus during functional range of motion as the “glenoid track” and found that Hill-Sachs lesions that extended medially over the glenoid track area during shoulder motion were at risk of engagement. These lesions were defined as off-track Hill-Sachs lesions and are associated with a higher degree of instability. Interestingly, it has been speculated that the glenoid track width may change depending on the shoulder’s range of motion [29]. As the horizontal extension angle increases, the glenoid track depth decreases, thus, increasing the risk of engagement. Clinical evidence supports the use of glenoid track in predicting postoperative stability. Shaha et al. [53] found that the glenoid track concept was a better predictor of risk of recurrence than isolated measurement of the glenoid defect. Similarly, Locher et al. [36] reported an 8.3-fold higher risk of failure of an arthroscopic Bankart repair in patients with off-track lesions than those with on-track Hill-Sachs lesions.

## Humeral head procedures

Procedures that directly address the humeral defect are usually indicated in patients with large and off-track Hill-Sachs lesions without or with a non-significant glenoid bone loss [60].

Bone humeral head augmentation can be performed with either bone grafts, synthetic materials or soft tissue (i.e. *remplissage*). The purpose of these procedures is to fill de humeral defect, thereby preventing the engagement of the lesion with the glenoid. Bone grafts include autograft iliac crest, or allografts, most commonly fresh humeral or femoral head allografts. Although usually performed in an open fashion, it has also been described as an arthroscopic technique. According to literature, less anterior translation with anterior load is achieved following this procedure [64]. However, concern exist about the high complication rate including graft resorption, graft failure, cyst formation and restriction of mobility [49]. Rotational humeral osteotomy has also been used for large humeral defects. In this technique the proximal humerus is transversely transected and derotated so that the lesion does not engage anymore [5]. However, high rates of complications including nonunion, over-rotation or fracture have been reported [49].

**Fig. 1:**
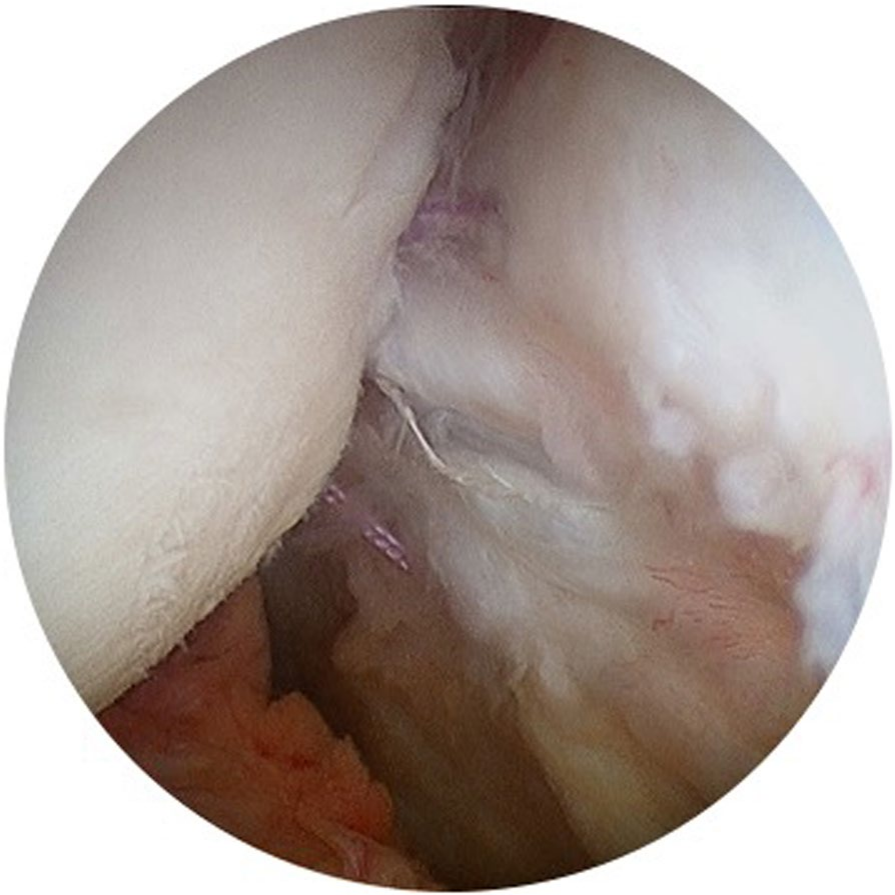
The *remplissage* procedure. Sutures passed through the infraspinatus and the posterior shoulder capsule are tightened filling the humerus defect

Conversely, soft tissue augmentation with the infraspinatus (*remplissage*) has shown good results and less morbidity than bone grafting procedures, so it is now a very common technique in the management of Hill-Sachs lesions [19]. As described in 2008 by Eugene Wolf, *remplissage* consists of filling the humeral bone defect by insertion of the lateral posterior shoulder capsule and infraspinatus tendon into de Hill-Sachs lesion [60] (Fig. 1). According to biomechanical studies, the *remplissage* procedure prevents off-track Hill-Sachs lesions engagement on the anterior glenoid rim. Elkinson et al. [18] found in their cadaveric study that shoulders with a 30% Hill-Sachs lesion engaged if a Bankart repair without *remplissage* was performed. However, engagement was prevented in all shoulders when Bankart repair was performed with *remplissage*. These findings were confirmed some years after by Hartzler et al. [25]. In their 8-cadaveric study, engagement occurred in all off-track lesions after an isolated Bankart repair but did not occur when adding *remplissage*.

Clinical results support these biomechanical findings. Postoperative recurrence rate was reduced from 18 to 4% in patients who underwent Bankart repair with *remplissage* compared with those who underwent isolated Bankart repair in MacDonald et al. series [37]. Low recurrence rates in were further supported by the systematic review performed by Camus et al. analyzing both patients with on-track and off-track Hill-Sachs lesions [12]. The authors noted a 4.5-fold higher risk of recurrence after isolated Bankart repair when compared with Bankart plus *remplissage*, with recurrent instability of 14.8% in the isolated Bankart repair group vs 1.4% in the *remplissage* group. Additionally, long-term follow-up series demonstrated that stability is maintained over time, with redislocation rates ranging between 0 and 11.8% after a minimum 5-year follow-up [19].

In terms of return to sports, Garcia et al. [19] reported a return to sports rate of 95.5% at an average of 7 months postoperatively, but only in 41.4% of those involved in overhead throwing sports. Similar rates were reported by Lazarides [34] with a 90.7% return-to sport rate and 70.4% of patients practicing sport at the same or higher level by 24 months postop.

The complication rate of this procedure is very low [49]. However, there is concern regarding the loss of range of motion, particularly in external rotation. Biomechanical studies on cadavers have reported a decrease on external rotation after Bankart with *remplissage* compared with the non-injured side. However, joint stiffness after *remplissage* was comparable to Bankart repair alone [18]. Moreover, clinical studies have found that this loss did not negatively affect shoulder function. Macdonald et al. reported a 10° decrease on external rotation after a *remplissage* procedure at 12 months [37]. However, this loss of range of motion did not significantly affect patient-reported outcomes or even return to sports. Furthermore, by 24 months, patients with and without *remplissage* had equal range of motion.

## Glenoid bone augmentation procedures

Glenoid bone augmentation is the primary method of managing significant glenoid bone defects [11]. According to the algorithm proposed by DiGiacomo [15], a remplissage procedure should be indicated to address those Hill-Sachs lesions without or little glenoid bone loss. However, this paradigm has been questioned and the Latarjet is now also indicated like *remplissage* in patients with large Hill-Sachs defects in the setting of recurrent glenohumeral instability [4, 13]. Although these procedures do not address the humeral head directly, they increase the articular glenoid surface, thus preventing engagement of the Hill-Sachs lesion. Glenoid augmentation could be either performed transferring the coracoid (Latarjet) or with free bone grafts, including iliac crest, distal tibia allograft, distal clavicle or scapular spine autograft.

### Latarjet

First described by Michel Latarjet in 1954, in this technique the coracoid process is transferred to the anteroinferior glenoid rim with the attached conjoined tendon. The stabilizing mechanism of this procedure is a combination of a “bone-block effect” obtained by the extension of the glenoid articular arc, and a “sling effect” produced by the tensioning of the transferred conjoined tendon in the subscapularis, particularly in abduction and external rotation [47].

**Fig. 2:**
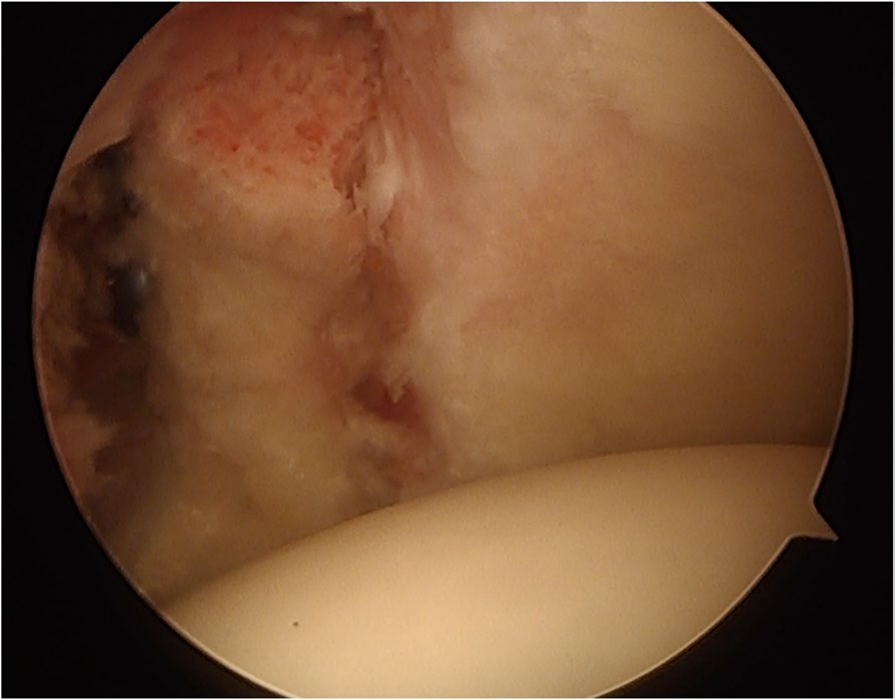
Intraoperative imaging of a Latarjet procedure with the coracoid graft accurate positioned

Since its description, different modifications have been proposed. In 2007, Lafosse et al. described the technique of an arthroscopic Latarjet procedure [33] which combines the benefits of the Latarjet procedure with the advantages of arthroscopic surgery (Fig. 2). Also, modifications in graft position and fixation have been described. Fixation was initially carried out by one screw and is now commonly performed with two screws. More recently, cortical buttons fixation devices have been introduced with satisfactory clinical and radiological results [3]. Regarding graft position, the congruent arch technique consisting of orientation of the coracoid with its inferior aspect congruent with the face of the glenoid has been suggested. The aim was to reproduce the curved anatomy of the glenoid articular surface. Normalization of glenohumeral contact pressures has been reported using this modification [1].

Overall, studies that examine the outcomes of the Latarjet procedure have reported good to excellent outcomes in both the open and arthroscopic approaches. Re-dislocation rate ranges between 0–8% after the open Latarjet technique according to Bhatia’s et al. systematic review [1]. Very low re-dislocation rates, noted at 2% have also been reported after an arthroscopic Latarjet procedure [23]. Functional outcomes and return to sport rates are also excellent [3].

Accurate position of the graft is obtained with both the open and arthroscopic techniques. Comparative studies between two techniques report satisfactory results and no differences between the two approaches [30]. Regarding graft union, the healing rate ranges between 66–89% [51] following an open technique and 73–95% [3, 40] in the arthroscopic group.

Despite the good clinical and radiological results reviewed above, several complications have been reported. Systematic reviews performed by Butt et al. [7] and Griesser et al. [22] reported a rate of complications up to 30%, including minor and major complications. A lower complication rate, noted at 7%, has recently been reported in the systematic review performed by Hurley et al. [26]. Similarly, a recent multicenter study of 1555 patients that had undergone an arthroscopic Latarjet procedure reported a 2.2% rate of major complications [23], including fracture of the coracoid graft [40], neurologic injury, especially injury to the axillary, musculocutaneous or suprascapular nerve [22], complications related to the fixation devices [7] and restriction of range of motion, especially external and internal rotation. On the other hand, both open and arthroscopic Latarjet require a split of the subscapularis muscle, which may lead to damage and disfunction of the muscle, although recent studies have found no differences in subscapularis function between injured and healthy side were observed at 2-year follow-up [59]. In addition, since it is a non-anatomic procedure, high risk of osteoarthritis exists, noted at 20% after a minimum follow-up of 20 years [41]. Finally, it is important to note that the arthroscopic approach is a complex and challenging surgical technique and caution should be paid to its learning curve [35].

**Fig. 3:**
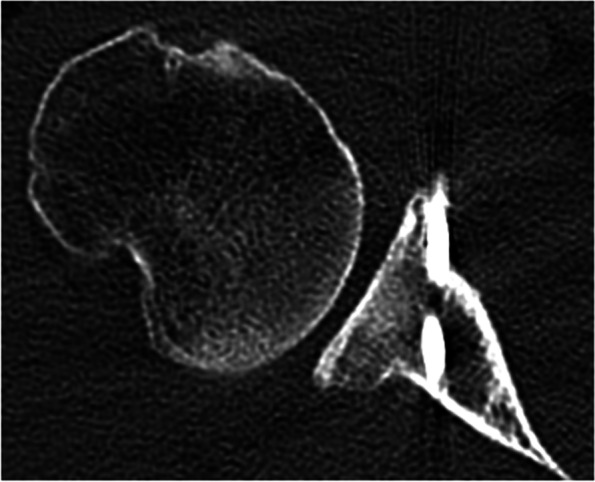
Postoperative CT-scan of a patient with a large and medial Hill-Sachs lesion that was not fully restored with the Latarjet procedure

Besides these complications, there are concerns on whether the Latarjet procedure can convert all off-track HS lesions into on-track lesions, especially in patients with bipolar glenohumeral bone loss or large bone defects (Fig. 3). Moon et al. [42] and Paladini et al. [45] both assessed whether the Latarjet procedure can fully restore the surface area of the glenoid in patients with large glenoid rim defects. 44 patients with a mean glenoid defect of 25.3% ± 6% and 143 patients, mean bone loss 26 ± 3.9% were respectively analyzed. Glenoid surface area was successfully restored in all patients. However, in a cadaveric study, Patel et al. [46] found that Hill-Sachs lesions greater than 31% were not sufficiently stabilized by the Latarjet procedure. The clinical study performed by Calvo et al. [9] supports this finding. In their study, 6 out of 51 (11.8%) Hill-Sachs lesions remained off-track despite the Latarjet procedure. The authors identified a Hill-Sachs interval wider than the glenoid track in a value greater than 7.45 mm as a risk factor for a persistent postoperative off-track lesion. Furthermore, the authors noted that persistent postoperative off-track lesions show a higher recurrence rate at a 24-months follow-up and recommend preoperative measurement of the glenoid track and the size of the coracoid process to confirm that the coracoid process is able to convert the off-track Hill-Sachs lesion into on-track. In these specific population of patients with large or very medial Hill-Sachs off-track defects another procedure should be added to the coracoid transfer, either concomitant bone grafting of the Hill-Sachs defect or *remplissage* [28, 50]. Larger free bone block procedures able to restore the glenoid track can also be recommended in this setting.

### Free bone graft procedures

**Fig. 4:**
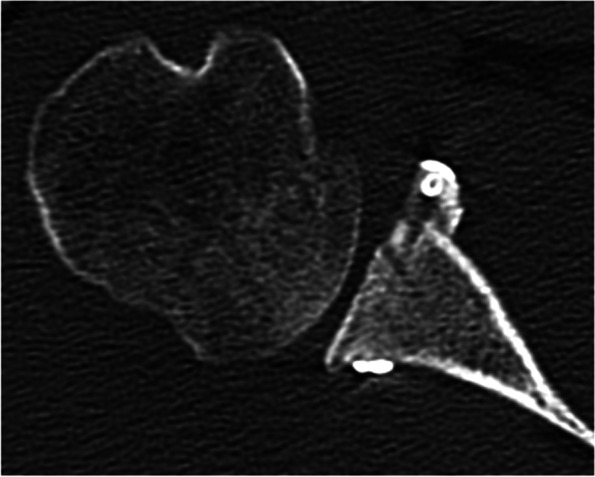
Postoperative CT-scan of a patient with an off-track Hill-Sachs lesions who underwent an arthroscopic Eden-Hybinette procedure. Courtesy of Dr Miguel García Navlet, Hospital Asepeyo Coslada (Madrid)

The first reports detailing an open free bone-grafting procedure to address glenoid bone defects were described by Eden in 1918 and Hybinette in 1932 [17, 27]. Arthroscopic bone-block procedure was developed by Scheibel [52] in 2007 (Fig. 4). The arthroscopic Eden-Hybinette procedure provides an anatomical reconstruction, preserves the integrity of the subscapularis tendon, and theoretically decreases the risk of damaging to neurovascular structures associated to the Latarjet procedure [52]. From a biomechanical standpoint, the theoretical limitation of free bone graft procedures when compared to Latarjet is the lack of the sling stabilizing effect provided by the conjoint tendon. Yamamoto et al. demonstrated in a biomechanical cadaveric study that the main stabilizing mechanism of the Latarjet procedure was the sling effect at both the end-range and the mid-range arm positions [63].

The Eden-Hybinette procedure has been suggested as a salvage procedure after a failed Latarjet procedure [39], but also as a primary surgery in patients with subcritical bone loss and reparable soft-tissues [56] or those with large bone defects not amenable to be reconstructed with the coracoid process bone graft [58].

Since its initial description the technique has suffered many modifications: the changeover from open surgery to arthroscopy, use of special instruments and glenoid guides to improve graft positioning [56] and development of different fixations devices besides screws, such us round-buttons [56] or sutures [24]. Also, different grafts have been proposed. Since Hybinette first harvested the iliac crest graft for this procedure, this area has remained the main autograft donor site. However, complications have been reported in up to 25% of the cases, including injury to the lateral femoral cutaneous nerve, hematoma, infection or persistent pain at the donor area [16]. The use of allografts may reduce these complications. Several sources of allografts have been used to address glenohumeral instability, including iliac crest [56] distal tibia [48] femoral head, and humeral head allografts [49]. However, allografts could have adverse effects, mostly risk of disease transmission or graft resorption [18]. Scapular spine and distal aspect of the clavicle have recently emerged as possible alternatives for reconstruction of the anterior glenoid rim [57, 61]. These grafts have the advantages of autologous grafts with less donor-site morbidity.

This technique provides excellent clinical results according to literature. A 2020 systematic review including 9 studies, 261 patients who underwent and Eden-Hybinette procedure (78% using iliac crest autograft and 22% iliac crest allograft), showed a recurrence rate of 4.8% [38]. Taverna et al. [56] reported a clinical series of 26 patients who underwent an arthroscopic bone-block procedure using iliac crest allograft. At two-year follow-up the Rowe mean score was noted at 94.6 and no redislocations were reported. However, it is important to note that not only patients with off-track Hill-Sachs lesion were included, but also those with on-track lesions and additional risk factors.

With regard to radiological results, arthroscopic bone-block procedure using specific guides provides accurate bone-graft positioning [14, 56]. Healing rates reported in literature vary widely [20, 65]. In a systematic review performed by Gilat et al. [21] analyzing the results of allografts and autografts iliac crest in the bone-block procedure, no differences were observed between the two groups with a mean healing rate of 78%. Regarding osteolysis, Kraus et al. [31] found resorption of the extraarticular part of the iliac crest autograft in patients with glenoid bone defects. With regard to allografts, significant rates of osteolysis were observed by Boehm in patients with iliac crest allografts [2]. Ten out of ten patients suffer a complete graft’s osteolysis 12 months after surgery. As a result, no restoration of the glenoid surface area was obtained. Distal tibia allografts to reconstruct the glenoid surface also show satisfactory results. Provencher et al. [48] in a case series of 27 patients, observed that 89% of the grafts were healed. Resorption occurred in 3% of the grafts. Promising results have also been obtained when using scapular spine autograft. In the study performed by Xiang et al. graft’s resorption was noted at 19.4% 1 year after surgery [61].

Studies comparing Latarjet and Eden-Hybinette procedure are limited. Moroder et al. [43] performed a randomized, controlled prospective study comparing clinical results at 24 months between an open Latarjet procedure and an arthroscopic bone-block technique in patients with recurrent anterior shoulder instability and glenoid bone loss. The authors reported no significant differences in WOSI index, patient-reported outcomes, and recurrence. However, complications were higher in the bone-block group, mostly resulting from harvesting bone graft. On the other hand, incidence of scapular dyskinesis and loss of internal rotation range of motion was higher in the Latarjet group when compared to iliac crest procedure. In 2020 Gilat et al. [21] published a systematic review comparing the outcomes between the Latarjet and Eden-Hybinette procedures. Seventy studies were included, and 4540 shoulders were evaluated. No differences in recurrence rate were found, noted at 5% in the Latarjet procedure and 3% in the Eden-Hybinette. This study did not find differences between the two procedures in complications rate, progression of osteoarthrosis and return to sports. Concerning Hill-Sachs off-track lesions, Eden-Hybinette has the advantage over Latarjet that the size of the bone graft can be tailored to restore glenoid track in patients with large or very medial off-track lesions [9]. Moreover, addition of *remplissage* to the Eden-Hybinette enhances stability, restoring stiffness closer to the native shoulder according to Callegari et al. [8].

## Conclusions

Off-track Hill-Sachs lesions can be managed with many different procedures. *Remplissage* has been proposed as a way of treating large and off-track Hill-Sachs lesions but little or no glenoid bone loss. Bone-grafting techniques have shown to be safe and effective in the management of bone loss and restoration of the glenoid track, thus preventing the Hill-Sachs lesion from engaging. Nevertheless, surgeons should be aware of a small subpopulation of patients with large or too medial humeral bone defects that could not fully be restored with the Latarjet procedure and may need either an Eden-Hybinette or adding additional techniques to the Latarjet such as a *remplissage*.
